# Force and Microstructure Variation of SLM Prepared AlMgSc Samples during Three-Point Bending

**DOI:** 10.3390/ma15020437

**Published:** 2022-01-07

**Authors:** Daming Nie, Ruilong Du, Pu Zhang, Fangyan Shen, Jason Gu, Yili Fu

**Affiliations:** 1 Interdisciplinary Innovation Research Institute, Zhejiang Lab, Hangzhou 310000, China; niedaming@zhejianglab.com (D.N.); zhangpu@zhejianglab.com (P.Z.); 2 Department of Electrical and Computer Engineering, Dalhousie University, Halifax, NS B3H 4R2, Canada; jason.gu@dal.ca; 3 State Key Laboratory of Robotics and System, Harbin Institute of Technology, Harbin 150000, China; meylfu@hit.edu.cn

**Keywords:** SLM, additive manufacturing, bending deformation, grain orientation

## Abstract

Lightweight parts manufactured by metal selective laser melting (SLM) are widely applied in machinery industries because of their high specific strength, good energy absorption effect, and complex shape that are difficult to form by mechanical machining. These samples often serve in three-dimensional stress states. However, previous publications mainly focused on the unidirectional tensile/compressive properties of the samples. In this paper, AlMgSc samples with different geometric parameters were prepared by the SLM process, and the variation of force and microstructure during three-point bending were systematically investigated. The results demonstrate that the deformation resistance of these samples has good continuity without mutation in bending, even for brittle materials; the bending force-displacement curves exhibit representative variation stages during the entire bending process; the equivalent bending strength deduced from free bending formula is not applicable when compactability is less than 67%. The variations of grain orientation and size of the three representative bending layers also show regularity.

## 1. Introduction

SLM manufactured parts occupy widespread application because of light density [1,2,3] and complex modeling, which present challenges in mechanical machining [4]. For example, the SLM parts are manufactured into bracket connectors on the Airbus A350 XWB [5] and could also be designed as cooling channels with optimized mechanical and heat dissipation performance [6], which are employed in the combustion chambers or turbines of aircraft engine. Yan et al. [7] studied the effect of porosity of Ti alloy SLM parts on biocompatibility [8] and proved that they possess equivalent compressive strength to human trabecular bones.

In this paper, AlMgSc was selected as the material of SLM parts. The microstructural variation of aluminum alloy at room temperature has been widely studied and reported [9,10,11]. Vu et al. [12] employed the process of Friction-Assisted Lateral Extrusion (FALEP) relative to Al-1050 alloy and successfully reduced the grain size more than 160 times down to 600 nm under the imposed shear strain of 20. The simple shear texture is obtained nearly parallel to the plane of the sheet. Rogachev et al. [13] analyzed the effect of high-pressure torsion (HPT) on the microstructure and tensile properties of the Al-10% La, Al-9% Ce, and Al-7% Ni alloys, showing the formation of nanocrystalline and submicrocrystalline structures and the refinement of eutectic particles in aluminum alloys. Yang et al. [10] investigated the cryogenic rolling (CR) process of T3003 aluminum alloy. The results demonstrate that this process can significantly decrease the size of sub-grains and second-phase particles and increase dislocation density. The initial Cube and R-Cube textures are gradually rotated into β-fiber texture. Moreover, a significant enhancement was detected in the yield strength and elongation of the alloy after the CR process.

S_C_ elements can precipitate from the Al-Mg matrix and form uniformly distributed nanoparticles on the grain boundary with the help of appropriate heat treatment parameters [12]. An Al_3_Sc lattice formed in aluminum matrix has a L1_2_ structure that also belonged to fcc structure, while the side length of the crystal cell is diverse. This attribution arouses strong and stable lattice distortion for hindering dislocation movement [14], thus significantly improving the mechanical properties and reducing the average diameter of grain size of the samples processed by SLM [15]. Some researchers added Zr, which has a similar action mechanisms relative to S_C_, as the substitute for S_C_ to reduce the cost, and it obtained even more excellent mechanical properties than those of high Sc alloys by using appropriate heat treatment [12]. Therefore, the mechanical properties of AlMgSc are not only related to the amount of added elements but also affected by the quality of element powder, scanning speed, hatch length, heat treatment process [15], etc. According to the report from Spierings et al. [16,17], Al_3_Sc particles precipitate only at low scanning speed due to relatively high temperature and long heating time or precipitate through the heat transfer of the subsequent layers. Due to mechanism, scanning speed further affects grain size; by doubling the scanning speed, the diameter of fine grains reduced from 1.1 μm to 600 nm, resulting in lower energy input. However, the size of coarse crystal has little variation [16]. These influence laws are sometimes not unified and need to be analyzed in combination with specific conditions [17].

At present, a large number of analyses about mechanical properties of SLM parts are reported; however, most of them are carried out in unidirectional tensile or compressive deformations [18,19], and few reports focus on multidirectional stress [20]. In practical applications, the parts mostly bear external force in multiple directions [21]. In this paper, the “plate-grid-plate” sandwich structures were manufactured by SLM processes, in which the grid layer is the main component, and the variations of the mechanical properties and microstructure are analyzed in characteristic deformation stages of the grid structures under three-point bending.

## 2. Experimental Methods

### 2.1. Material and Structural Dimensions

**Figure 1:**
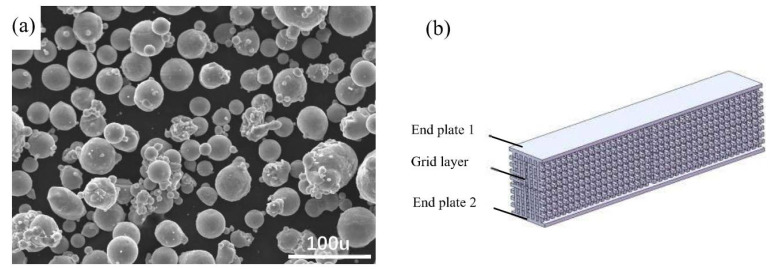
Material and structure of specimens: (**a**) AlMgSc alloy powder after atomization spray forming; (**b**) schematic diagram of specimen model.

The material of the specimen is AlMgSc, with the composition of Mg wt. 2.22%, Al wt. 96.96%, and Sc wt. 0.82%. Al, Mg, and Sc were melted and gas atomized to form alloy particles with 2.5 MPa gas pressure (Figure 1a), which is taken as the powder of laser selective melting process. In terms of powder quality, the ratios of deformity and adhesion are 8.4% and 15.8%, respectively, and the average particle size is 30 μm, which is similar to the report by Bayoumy et al. [22]. The specimen possesses sandwich structure, for which its upper and lower end layer comprise a continuous thin plate with a thickness of 1 mm, and the middle layer is grid structure [23] with a thickness of 18mm (Figure 1b). This thickness ratio (1:18:1) is chosen because it is often utilized in practical applications.

The dimension of the specimen is 240 mm long, 14 mm wide, and 20 mm thick, with SLM power of 335 W, speed of 1000 mm/s, and powder-layer thickness of 30 μm. In order to analyze the influence of size and orientation of the struts, samples with different geometric parameters are designed and illustrated in Table 1. Multiple samples are prepared at the same time to improve efficiency during manufacturing.

**Figure 2:**
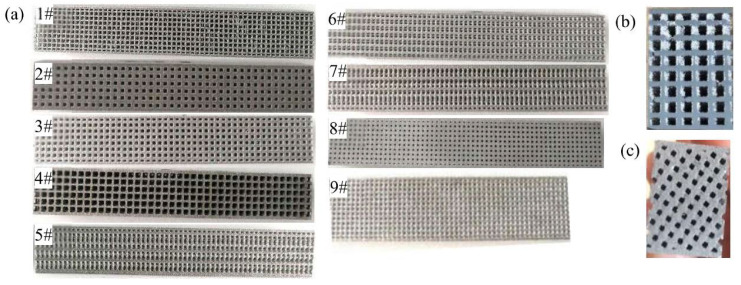
Samples manufactured by SLM: (**a**) samples with different geometric parameters, cross sections of (**b**) 2#, and (**c**) 7# sample.

Nine groups of specimen with different geometric parameters are acquired (Figure 2) and numbered in sequence of compatibility from small to large. It can be observed from (b) and (c) that the grids of the samples are clear and the dimensional accuracy is high, manifesting outstanding forming performance of AlMgSc material.

### 2.2. Three Point Bending

**Figure 3:**
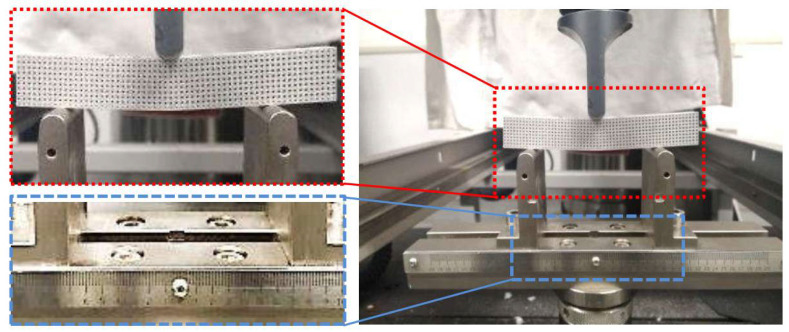
Three point bending device of SLM specimen.

**Figure 4:**
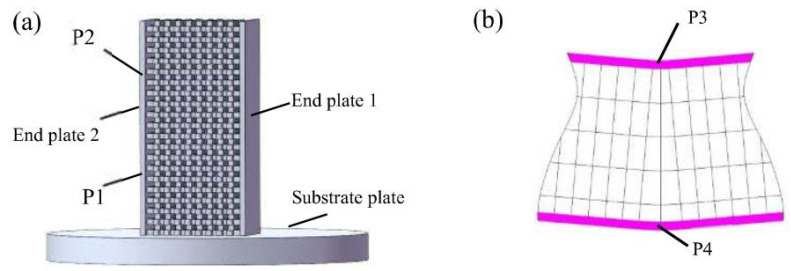
Schematic diagram of EBSD specimen: (**a**) unbent sample; (**b**) bent sample.

Instron 5982 testing machine was selected for three-point bending, as shown in Figure 3. The maximum tensile force of the equipment is 100 kN, and the displacement measurement accuracy is 0.05%. This configuration meets the test requirements of this experiment. The bending punch is set to be aligned with the symmetry axis of the specimen. The round corner diameter of the punch is 20 mm, the bending span is 160 mm, the pressing speed of the punch is 2 mm/min, and the entire bending process is recorded in video to observe the fracture process. The points on the center line parallel to the width direction on the upper surface of the specimen (P3 in Figure 4b observed in width direction) have the same Z-direction displacement as the punch; thus, their displacement is regarded as the bending displacement. For the sake of eliminating random errors, three experiments were repeated for each experimental group.

### 2.3. Fracture Analysis

The SEM photographs taken by Zeiss sigma 300 are employed to observe the fracture planes of the specimen after bending. The fracture position of the upper and lower end plates and intermediate struts (M) of the specimen are selected as the testing spots. For comparison, three of the nine groups of samples, i.e., 2#, 4#, and 8# in Section 2.1 were employed to explore the diverse of fracture morphology determined by the thickness of the struts. In order to improve detection efficiency, three samples are placed into the sample chamber for simultaneous detection.

### 2.4. EBSD Testing

The EBSD images taken by Hikari XP of EDAX are adopted to analyze the size and grain orientation of the specimen. Four representative spots in two groups of scanning area are employed; the two spots in first group are located in 1/3 height from the substrate plate (**P1** in Figure 4a) and 1/3 height from the upper end before bending (**P2** in Figure 4a), respectively. The other two spots in second group are the center of the bent upper and lower end plate of the sample (**P3** and **P4** in Figure 4b). The electric field voltage for scanning is 15 KV, the area of the field of view is 138 μm × 138 μm, and the scanning step is 0.15 μm. EBSD specimens were prepared by electrolytic polishing with a solution ratio of phosphoric acid: sulfuric acid: chromic acid = 0.5:0.4:0.1.

## 3. Analysis of Experimental Results

### 3.1. Bending Fracture Analysis

**Figure 5:**
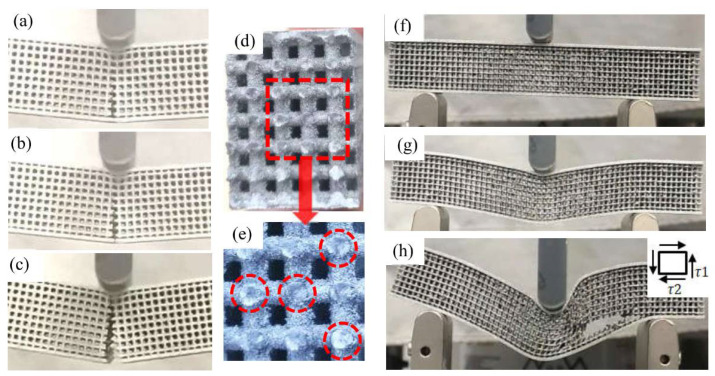
Bending process of samples. (**a**) initial stage of sample 2#–9#; (**b**) intermediate stage of sample 2#–9#; (**c**) late stage of sample 2#–9#; (**d**) fracture surface of sample 2#–9#; (**e**) enlarged view of the fracture surface; (**f**) initial stage of sample 1#; (**g**) intermediate stage of sample 1#; (**h**) late stage of sample 1#.

There are two modes of crack propagation during bending of the AlMgSc specimen. The first is illustrated in Figure 5a–c, the outer side of the grid layer breaks first, and the lower end plate begins to break after the crack reaches the middle position of the grid layer; subsequently, the crack gradually extends inward, and finally the upper end plate breaks. This fracture mode is applicable to 2#–9# groups. The maximum stress in the length direction concentrated in the adjacent of grid and lower end layer; thus, the specimen easily starts breaking at the outside of the grid layer and then the cracks propagate to the inner. Moreover, collapse occurs at the surface of the upper end plate in contact with the bending punch, which is similar to that of rectangular beam bending [24] resulting from the gap in grid layer and pressure caused by the punch.

The second mode of crack propagation is shown in Figure 5f–h for the 1# sample for which its ratio of diameter to unit length is 0.25; the stress-strain state in fracture position is shown in Figure 5h. It can be observed that the material is deformed along the length direction due to shear stress, and breaks at the joint of the lower end plate and the grid layer. The left and right sides should be deformed symmetrically under ideal conditions, and this is difficult achieve in practice.

**Figure 6:**
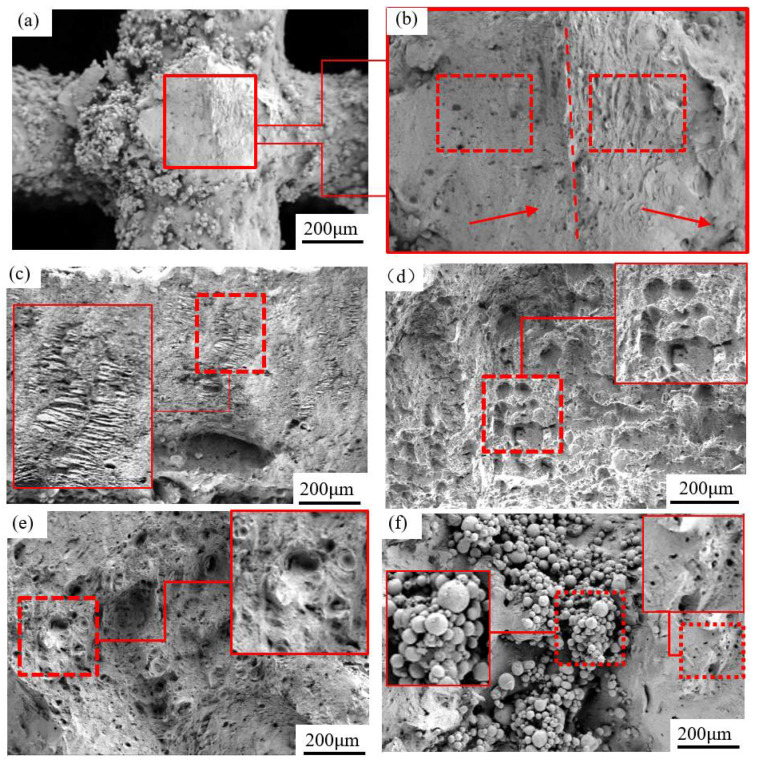
Fracture morphology of SLM specimen. (**a**) strut; (**b**) enlarged view of strut; (**c**) tensile band; (**d**) pores in sample with lower comatibility; (**e**) pores in sample with higher comatibility; (**f**) unmelted powder.

Figure 5d reveals the macro morphology of the fracture, which is flat and smooth for 2#–9# samples. Nevertheless, the fracture location and orientation are remarkably distinct in microscope (Figure 5e) even for neighboring struts due to inhomogeneous microstructure, which is also observed by Tian et al. [25]. Furthermore, there may be two orientations of fracture surface on a single strut (Figure 6a,b): The right one is a tensile zone with cleavage surface and the left one with pores is the compression zone. Moreover, tensile bands [26,27] appearing in the lower layer of bending indicate little local plastic deformation (Figure 6c).

The micromorphology of 2#–9# sample fracture is exhibited in Figure 6. The entire fracture is smooth, without dimples, which certificates the brittle fracture [28]. There are large numbers of unmelted powder particles on the fracture planes of the upper and lower end plates (Figure 6d), and the particles and surrounding voids are responsible for the brittle fracture [15,29]. The samples with higher compatibility display fewer unmelted powder particles (Figure 6e) on the sections due to more heat at the end plate during SLM forming transferred by much dense struts. Metal particles adhered on the surface of the struts [30], and the intensity increases with compatibility (Figure 6f). The cause for this phenomenon is that the unmelted particles were connected with the heated and melted ones nearby, inducing large connection strength after cooling. In addition, multilayers of powder particles are bonded together without pressure due to the adhering effect of the droplets generated by splashing at the unmelted powder layer. Shrinkage holes appear on the surface of struts (Figure 6f), resulting from volume shrinks when the liquid metal solidifies [31].

### 3.2. Analysis of Bending Curves

**Figure 7:**
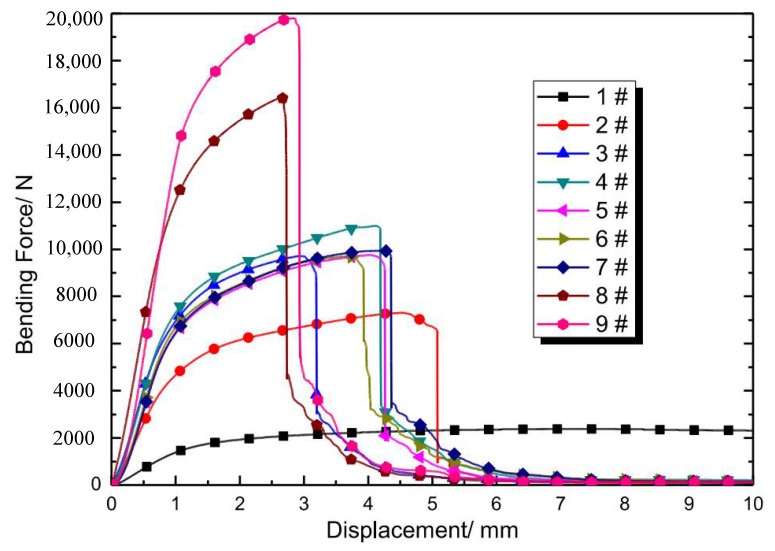
Bending force-displacement curves of specimens with different geometric parameters. The structural features of each coded sample are indicated in Table 1.

Several apparent phenomena could be concluded while analyzing the combination of the bending curves (Figure 7) and experimental fracture process:

(1) The curve trend is continuous without a step before total fracture, although the struts are broken successively during bending. The grid struts are brittle based on the morphology analysis of the cross section, and the “stress step” should appear at the moment, yet the curve is continuous here. This phenomenon may be attributed to two causes: the first is the inhomogeneous fracture as presented above, and the second is local plastic deformation, which could be confirmed by the tensile band in the fracture plane (Figure 6c).
(2) The sudden drop of the curve appears uniformly in the fracture of the upper end plate, which appears in the late stages of the entire deformation process. There is no reduction in deformation resistance, although the successive brittle fracture of grid struts during bending is observed. The sandwich structure is advantageous in such deformation modes. Even if the material is brittle, there will be no sudden drop of bending resistance before the fracture of the upper end plate.
(3) As illustrated in Figure 5h, the elongation and strength of 45° oriented struts are the lowest in three-point bending, while the differences between those of 30° and 60° struts are little. The cause is that the stress state of the 45-degree oriented strut is the most prone to shear deformation [32].

**Figure 8:**
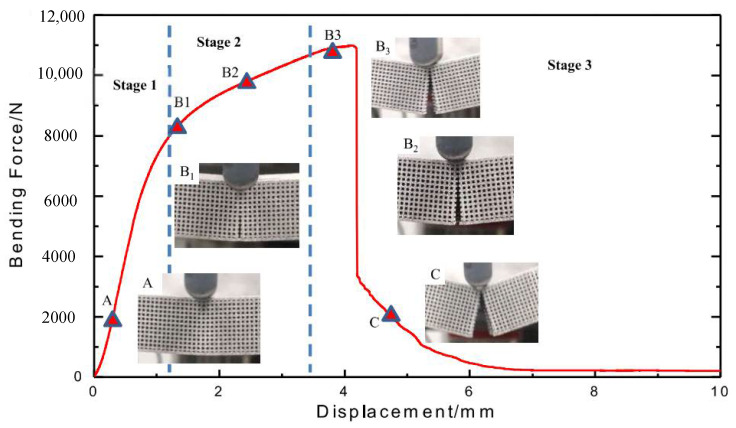
Different stages of specimen bending.

From the analysis of bending curves, the maximum bending force decreases with compactability while displacement increases in general, and the trend of the curve is similar to the unidirectional tension of elastoplastic materials. For the 1# sample, there is no sudden drop of force throughout the bending process, but a long plateau was observed; for 2#–9# samples, the deformation of each sample features three stages (Figure 8) regardless of the specific value difference of the bending curves: stage one is elastoplastic deformation [33] without macro crack in bending (A point); stage two is the initiation and expansion of the crack until the grid layer is completely broken (B1–B3 points); and the stage three is the fracture of the upper end plate (C point). This is similar to Jindra’s experimental results [34].

### 3.3. Equivalent Bending Stress Calculation

The classical mechanical formulas of three-point bending are generally treated approximate to that of the free bending, which is an ideal case. It requires several assumptions: (1) The strain changes along the radial linearly, (2) the cross section is successive without macroscopic hole, and (3) there is no pressure between bending layers. However, the width of the section, taking I-beam as an example [35], is inhomogeneous and, thus, could result in stress concentration. On the other hand, for the convenience of engineering calculation, the concentration is often ignored in mechanical analysis for the structures with large stiffness. Similarly to I-beam, the sandwich structures in this paper yielded stress concentrations in the middle layer, i.e., grid layer. Since the ideal assumptions mentioned above may not be met and may even be seriously deviated in some cases, the analysis of the applicability of the free bending formulas has important engineering value to the three-point bending of the sandwich structures.

For the three-point bending of continuum plates, bending stress σ could be expressed similar to that of free bending, as stated below [36]:

(1) $$\sigma =\frac{M_{x}}{I_{z}}y=\frac{\frac{Fl}{2}}{\frac{bh^{3}}{12}}y=\frac{\;6Fl}{bh^{3}}\;y$$

where *b* and *h* are the width and thickness of the specimen, respectively, *l* is the bending span, *F* is the bending force, $I_{z}$ is the moment of the bending inertia, $M_{x}$ is the bending moment, and *y* is the distance from the bending neutral layer.

The sandwich structure is symmetrical; thus, the horizontal center line plays the role of a neutral layer in bending. The bending outer layer is divided into two parts: the bent lower end plate and half of the space grid layer. The bending moment of the lower end plate is calculated consistently with the continuum plate, while the grid layer needs to be simplified. As mentioned above, thickness of the bent lower end plate is 1 mm, and the middle layer, namely the grid structure, occupies a thickness of 18 mm. Since the grid is discontinuous along three directions, there is no strict formula for the bending moment of inertia. Here, the method of multiplying continuous plate data by the compactability of the grid is estimated. The bending moment of inertia is stated as follows:

(2) $$I_{z1}=\int_{A}^{}y^{2}dA=\frac{b\left(H^{3}-h^{3}\right)}{12}$$

(3) $$I_{z2}=\int_{A}^{}y^{2}dA=\frac{\frac{b}{2}\left(h^{3}\right)\frac{1}{2}}{12}=\frac{bh^{3}}{48}$$

(4) $$I_{z}=I_{z1}+I_{z2}=\frac{b\left(H^{3}-h^{3}\right)}{12}+\frac{bh^{3}}{48}=\frac{4b(H^{3}-3h^{3})}{48}$$

where $I_{z1}$ and $I_{z2}$ are the bending moment of inertia of the continuum end plates and grid layer, respectively, A is the differential area of stress, y is the distance from the bending neutral layer, b is the width of the structure, H is the thickness of the sandwich specimen, and h is the thickness of the grid layer.

More generally, for the grid layer with strut length a and diameter *d*, the moment of inertia is calculated as follows.

(5) $$I_{z2}=\int_{A}^{}y^{2}dA=\frac{b\frac{d}{a}\left(h^{3}\right)\frac{d}{a}}{12}\;=\frac{bh^{3}}{48}{\left(\frac{d}{a}\right)}^{2}$$

Bending section coefficient $W_{z}$:

(6) $$W_{z}=\frac{I_{z}}{y_{max}}$$

Equivalent bending stress σ:

(7) $$\sigma =\frac{M_{z}}{W_{z}}=\frac{\frac{Fl}{2}}{I_{z1}+I_{z2}}y=\frac{\;Fly}{\frac{b\left(H^{3}-h^{3}\right)}{6}+\frac{bh^{3}}{24}{\left(\frac{d}{a}\right)}^{2}}$$

The bending displacement ρ, i.e., the bending radius of the specimen, can be calculated as follows:

(8) $$\rho =E\frac{y}{\sigma }$$

where E is the elastic modulus of the material.

**Figure 9:**
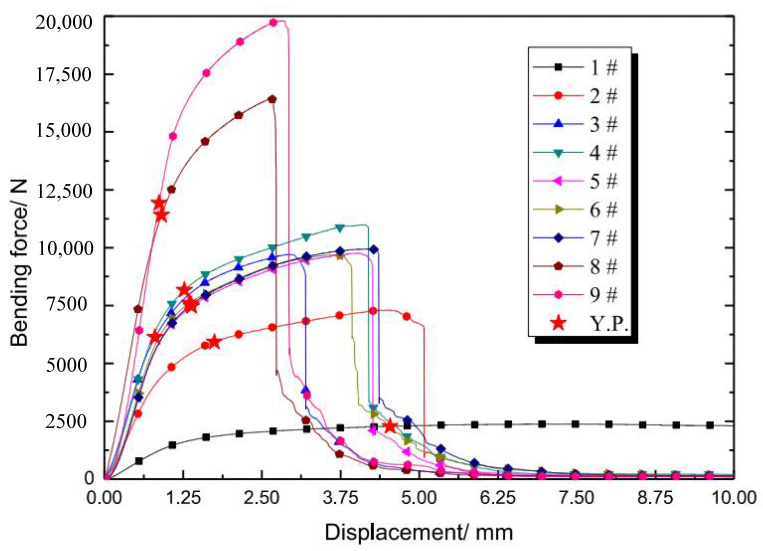
Early characteristic stages of bending. The structural features of each coded sample are indicated in Table 1.

In the elastic deformation stage, the stress is distributed in gradient form, and the surface bending stress of the lower end plate is the largest. This regulation is employed here to check the applicability of the free bending formula reversely. The checking procedure is detailed as follows: First, the yield strength of the material AlMgSc can be determined as 281 MPa based on our fundamental mechanical test; second, the bending displacement ρ can be calculated when the equivalent bending stress reaches the yield strength based on Equation (8); third, by comparing the calculated bending displacement and the data in experimental bending curve, the feasibility of the data could be judged. For instance, the compactability of the grid is 0.75 for 9# specimen, and bending displacement ρ is calculated as 0.86 mm according to Equation (8), thereby the corresponding data spot, i.e., the calculated yield point (Y.P.) in the bending curve of the 9# specimen is marked in Figure 9. This point is located in the transition position from elastic to plastic deformation; therefore, the calculated result may be reasonable. In a similar manner, the feasibility of 1#–8# specimens could be judged and the calculated yield points (Y.P.) are all marked on corresponding bending curves in Figure 9.

It could be concluded that the formula of free bending is not feasible for 1#–7# specimens, for which their compactabilities are below 0.67, due to the analysis of Figure 9. All of the calculated Y.P.s of 1#–7# specimens lie in stage 2, where the structures have deformed in plasticity and are cracked already; thus, the points calculated as Y.P.s are wrong. The calculated Y.P. belongs to the pure elastic stage and near the true critical point between the elastic and plastic deformations for 8# sample. The reason for the inaccurate judgment of 1#–7# samples is that compatibility is relatively small for these specimens and deviates seriously from the assumption of homogeneity relative to the free bending formula.

### 3.4. Microstructure Analysis

The features on the microstructure of the specimens during bending could be analyzed in two steps. The first step is to observe spots **P1** and **P2** mentioned in Figure 4 and the second step is analyzing the grain orientation in the longitudinal section of the bent upper (spot **P3**) and lower (spot **P4**) end plate.

**Figure 10:**
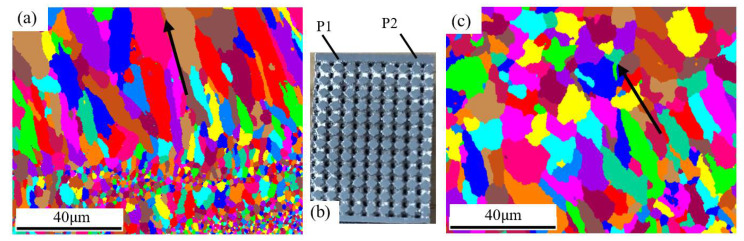
Grain structure on different spots: (**a**) spot P1; (**b**) P1 and P2 located in specimen; (**c**) spot P2.

**Figure 11:**
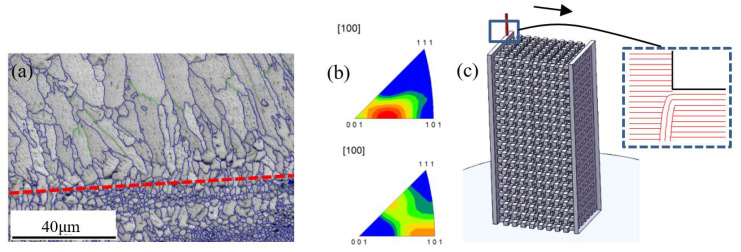
Grain structure of different parts: (**a**) first forming layer, (**b**) later forming layer, and (**c**) schematic diagram of forming process.

The results in the first step manifest both spots, including columnar and equiaxed grain regions (Figure 10a); the average diameters are 8 μm for the columnar grains and 3 μm for the equiaxed grain in spot **P1**, which is close to those obtained by Spierings [16,37], with a rapid cooling rate (10^6^~10^8^) [38]. The intergranular angle of most grain boundaries in the two regions is greater than 15° (blue line in Figure 11), which was also observed by Li et al. in Fe-Mo alloy [38]. The equiaxed grains are grown in the molten pool, and the columnar grains are located in the middle of the two neighboring layers of equiaxed grains, which belong to directional growth caused by heat transfer from the molten pool. In terms of grain size, the columnar grains at **P1** become smaller, and the equiaxed grains become larger (Figure 10c), indicating that heat flow affects the solidified region in two ways: one is the grain splitting in the columnar grains region and the other is the grain merging of the equiaxed fine grains. The intergranular angle of the grain boundary is still greater than 15° after recrystallization, which suggests that further recrystallization is possible [39].

The grains have intensive [103] fiber texture along the length direction (Figure 11b) in region P2. Compared with the fiber texture near the substrate plate, the grain orientation transfers from [103] to [101], indicating that the intensive heat flow could result in certain deflection of direction to [103] on the basis of [101] at the beginning of laying. With an increase in laser scanning slices, heat transfer tends to be perpendicular to the scanning direction in critical conditions; hence, grain orientation is revised to [101].

**Figure 12:**
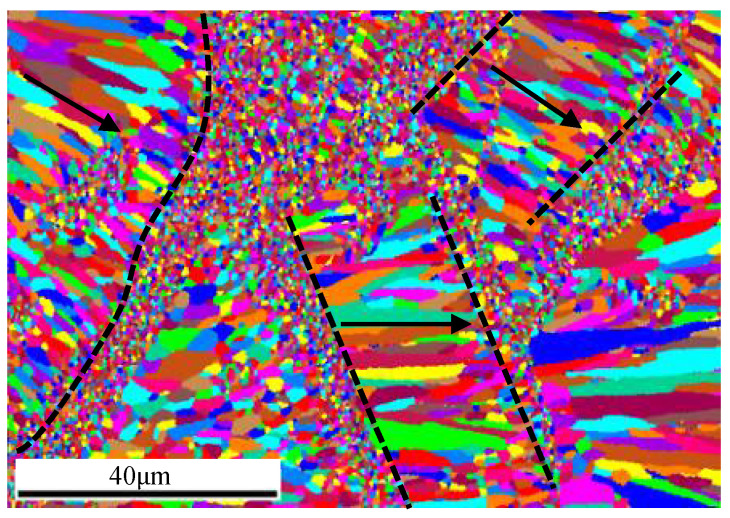
Microstructure variation in different paths.

In addition, the scanning paths are not carried out in one direction during the manufacture of sandwich structures, which may cross each other and produce columnar grains in multiple orientations in local regions, as shown in Figure 11c and Figure 12. Subsequent melting reconstructs previous melting regions, and the orientation becomes complex.

For step two of the investigation, grain orientation is analyzed in the longitudinal section of the bent upper (spot **P3**) and lower (spot **P4**) end plate. During bending deformation, grain breakage appears in the local columnar grains region similar to rolling [40], forming equiaxed crystals for which their grain size is larger than that of the original equiaxed grain region. The breakage occurring in specific regions demonstrates that breakage occupies priority directions. The stress state of the upper plate is different from that of the lower one; the upper end plate is compressed first and then tensioned during bending. Therefore, the broken grains are thinner than those simply stretched in the lower end plate of bending.

**Figure 13:**
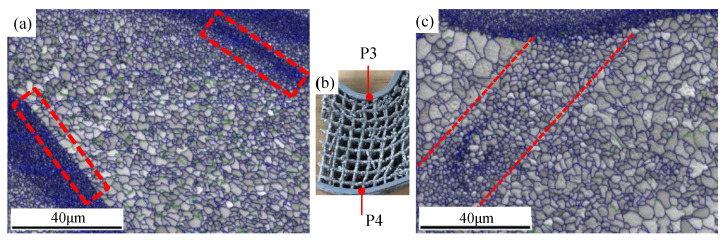
Grain distribution in different spots after bending: (**a**) characteristic regions of grain distributioin; (**b**) the detected spots; (**c**) crushed grains during bending.

The variation of grain size is diverse between the two frontiers of the equiaxed grain region (Figure 13), and grain size alternation at one frontier is distinct while moderate at the other. The cause is that slow-transited grains develop with the help of heat conduction, which is in accordance with the temperature gradient. The precipitation of triangular or square Al_3_Sc particles [40] is responsible for distinct changes of grain size in the other frontier regions; the grain is small due to the pinning effect of Al_3_Sc.

**Figure 14:**
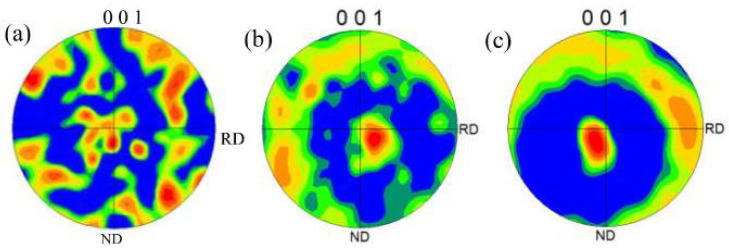
(001) Pole figures: (**a**) unbent; (**b**) bent lower end plate; (**c**) bent upper end plate.

The grain orientation variations of the characteristic positions after bending are regulated as follows: The (001) grain orientation of the lower end plate is more chaotic than that of the upper one (Figure 14). The original orientation is weak due to the low Hall–Pitch coefficient for the aluminum matrix [4]. The texture of the bent upper end plate on width direction is stronger than that of the lower one. This phenomenon may be due to the deformation state of compressing first and stretching afterwards in the upper end plate, which causes the (001) crystal plane to rotate towards the transversal direction, namely more parallel to the width direction.

The explanation of smooth transformation of the bending curves in Figure 7 could be partly given by the microstructure analysis conducted here. The change of grain size and orientation (Figure 13 and Figure 14) by crushing may promote plastic deformation and greater strength along the length direction. Therefore, no sudden change occurs in deformation resistance, although the strut’s fracture is mainly brittle.

## 4. Conclusions

In this paper, the samples with diverse geometric parameters were manufactured by SLM process, and the mechanical behavior and microstructure variation regulations of samples in three-point bending were investigated. The following conclusions could be drawn:

1. The samples mainly fracture in a brittle manner during bending, and the force varies continuously with displacement. The causes include the inhomogeneous breakage of the struts in the grid layer and the plastic deformation realized by grain crushing and rotation in bending.
2. The change rate of stress in three-point bending of the samples demonstrates three characteristic stages: in the first stage, the change rate increases with displacement due to rigid movement; the change rate in the second stage remains constant, which denotes elastic deformation; and in the third stage, the change rate decreases with an increase in displacement, which includes plastic deformation.
3. The calculation method of equivalent bending stress in free bending is not applicable to sandwich structure samples when compactability is below 67% due to heavy deviation to ideal assumptions.
4. The heat generated by the late-forming layer causes recrystallization of the early forming layer; the grain size becomes larger in the equiaxed grain region, and [101] crystal orientation is distributed perpendicularly to the scanning direction.
5. The (001) texture of the bent upper end plate on width direction is stronger than that of the lower one, which may be due to the compression–tension deformation mode.

## Figures and Tables

**Table 1:** Samples with different geometric parameters.

Number	Unit Length (mm)	Strut Diameter (mm)	Compactability	Inclination (Degree)
1#	2	0.5	0.25	0
2#	2.5	1	0.4	0
3#	2	1	0.5	0
4#	3	1.5	0.5	0
5#	2	1	0.5	35
6#	2	1	0.5	45
7#	2	1	0.5	60
8#	1.5	1	0.67	0
9#	2	1.5	0.75	0

## Data Availability

The datasets used and/or analyzed during the current study are available from the corresponding author on reasonable request.
